# *Galium shinasii* (Rubiaceae): a new species of *Galium* L. from Eastern Turkey

**DOI:** 10.3897/phytokeys.75.10244

**Published:** 2016-11-29

**Authors:** Levent Şık, Hasan Yıldırım, Ademi Fahri Pirhan, Yusuf Altıoğlu, Meliha Gemici

**Affiliations:** 1 Department of Biology, Faculty of Science and Arts, Celal Bayar University, 45030 Manisa, Turkey; 2 Ege University, Faculty of Science, Department of Biology, 35100, Bornova-Izmir, Turkey

**Keywords:** Rubiaceae, Galium, ecology, taxonomy, Turkey

## Abstract

*Galium shinasii* Yıldırım (Rubiaceae), is described as a new species from Malatya Province in eastern Anatolia, Turkey. The new species is morphologically related to *Galium cornigerum* Boiss. & Hausskn. *Galium lasiocarpum* and *Galium sorgereae* Ehrend. and Schönb. but clearly differs from them based on the morphological differences presented in the species description. In addition, the conservation status, the distribution map, and notes on the biogeography and ecology of the new species are given.

## Introduction

The family Rubiaceae is the fourth-biggest angiosperm family with about 660 genera and 11.500 species (Robbrecht and Manen 2006, Soza and Olmstead 2010). A large number of species of Rubiaceae are herbaceous and many are adapted to xeric habitats. (Robbrecht 1988, Jansen et al. 2000). *Galium* L. is one of the largest genera of the Rubiaceae, with about 650 species and approximately 780 taxa placed in 16 sections, including perennial and annual herbs that are distributed in temperate and tropical regions of worldwide (Willis 1985, Mabberley 1987, Goavert 2015).

A total of 121 *Galium* taxa (104 species) are found in Turkey and 60 taxa (endemism rate of 50%) are endemic to this country (Karabacak 2012).

Malatya is located in the eastern part of Turkey, one of the richest centres of species endemism in Turkey (Yıldız et al. 2004) and in recent years several new species have been described from this area and still continue to be discovered in Malatya (Yıldırım et al. 2010, Mutlu and Karakuş 2012, Tan et al. 2012, Koç and Aksoy 2013, Yıldırım and Erol 2013, Yıldırım and Şenol 2014a, 2014b, 2014c, Mutlu and Karakuş 2015a, 2015b, Yıldırım 2015a, 2015b).

Levent Canyon is one of the famous areas among biologists because of the high endemism in Malatya province. Recntly, several new plant species were described in this area. Levent Canyon is characterized by marlstone, a soft, finely fissured sedimentary rock (Schnurrenberger et al. 2003, Yıldırım and Şenol 2014c) which hosts many chasmophytes. The Levent Canyon is also a centre of diversity and endemism for several chasmophyte species. For example, *Reseda malatyana* Yıldırım (Resedaceae) and Şenol, *Alkanna malatyana* Şenol and Yıldırım (Boraginaceae), *Campanula alisan-kilincii* Yıldırım and Şenol (Campanulaceae), *Galium scopulorum* Schönb.-Tem. (Rubiaceae), *Pimpinella paucidentata* V.A.Matthews (Apiaceae), *Parietaria semispeluncaria* Yıldırım (Urticaceae) and *Galium cornigerum* Boiss. and Hausskn (Rubiaceae).

In June 2011, the second author collected an unusual and distinct specimen of *Galium* on marlstone-calcareous rocky cliffs in the Levent Canyon which authors believe to be of a new species for science.

## Materials and methods

Specimens of the putative new species were compared with herbarium specimens at ANK, EGE, E, G, GAZI, HUB, ISTE, K, W and WU. In addition, the relevant literature (Halácsy 1901, Ehrendorfer and Krendl 1976, Ehrendorfer and Schönbeck-Temesy 1982, Davis et al. 1988, Ehrendorfer and Schönbeck-Temesy 2005, Govaerts 2016) was reviewed. The new species was examined by stereo-binocular microscope for morphological characterisation. At least 20 mature seeds and 30 pollen grains were measured using a light microscopy. For scanning electron microscopy (SEM), the selected seed and pollen grains were placed on aluminum stubs using double-sided adhesive tape, sputter coated with gold using an Emiteck K550, and then examined using the FEI Quanta250 FEG SEM. Photographs of living material were taken with a Nikon D300 digital camera. The conservation status of new species was evaluated based on the field observations in accordance with IUCN guidelines (2012). Geographical positions were recorded using a Magellan explorist 500 GPS.

## Results

### Galium shinasii

Taxon classificationPlantaeGentianalesRubiaceae

Yıldırım sp. nov.

urn:lsid:ipni.org:names:77158796-1

Figure 1 , 2 , 3

#### Type.

Turkey: B7 Malatya: Akçadağ district, Levent Canyon, on marlstone rocky cliffs 1390 m, 26.06.2011, *H.Yıldırım 2128* (holotype: EGE42431!, isotypes: EGE42432!, NGBB!, ANK!).

#### Paratype.

Turkey: Malatya: Akçadağ district, Levent Canyon, on marlstone rocky cliffs 1390 m, 29.06.2015, H.Yıldırım 3358 (EGE!) (Figure 2A); loc., ibid., 11.09.2015, H.Yıldırım 3713(EGE!); Doğanşehir, Eskiköy, Meletbaşı mezrası karşısı kalker kayalıklar, 1630–1800 m., 12.07.2014, H.Yıldırım 3033 (EGE!) (Figure 2B). Erzincan: Sivas-Refahiye yolu, Refahiye’ye 1–2 km kala, kalker kaya üzeri, 1528 m, 09.09.2015, H.Yıldırım 3694 (EGE!) (Figure 2C).

#### Diagnosis.

*Galium shinasii* is related to *Galium cornigerum*, *Galium lasiocarpum* and *Galium sorgereae* but it differs from them in having very reduced flowers (not flowers relatively larger), 1.2–1.8 mm corolla diam (not 2–5 mm); yellowish-green to reddish-green and 0,5–1 mm long tepals (not white or pink and not at least 2 mm); dorsal and ventral surface of with densely transparent tubercles and lateral surface 0.2–0.4 mm spreading to patent hairy fruits (not tubercles absent and fruits wholly villous, hirsute or subtomentose).

#### Description.

Dwarf, caespitose perennial plant with many headed rootstock, suffruticose at base. Stem 1.5–6 cm long, fragile, prostrate-ascending to erect, many branched at base, glabrous to slightly puberulent, sometimes slightly winged on nerves, upper internodes elongate to 5 mm. Leaves in whorls of mostly 4, rarely 6, linear-lanceolate to narrow elliptic, 2–8 × 0.6–1.3 mm, 1 veined, glabrous to slightly puberulent, revolute at margin. Inflorescense dicashium, mostly terminal and also axillary, 8 to 75 flowered per stem; bracteoles absent. Pedicel glabrous, 1.5–2.5 mm in flowers, 2–5 mm in fruit. Calyx absent. Corolla 4 merious, yellowish-green to reddish-green, 1.2–1.8 mm diam; usually conical or campanulate, rarely infundibular; tube very reduced; lobes 0.5–1 × 0.4–0.7 mm, glabrous, triangular to lanceolate, mucronate at apex and apex incurved on petal inner surface. Stamen 0.4-0.6 mm long; anther yellow. Ovary 0.4–0.5 mm diam, dorsal and ventral surface of with densely transparent tubercles, lateral surface 0.2–0.4 mm spreading to patent hairy. Fruit depressed subglobose in fleshy, 0.5–0.75 mm, dorsal and ventral surface of with densely transparent tubercles, lateral surface 0.2–0.4 mm spreading to patent hairy.

**Figure 1.**
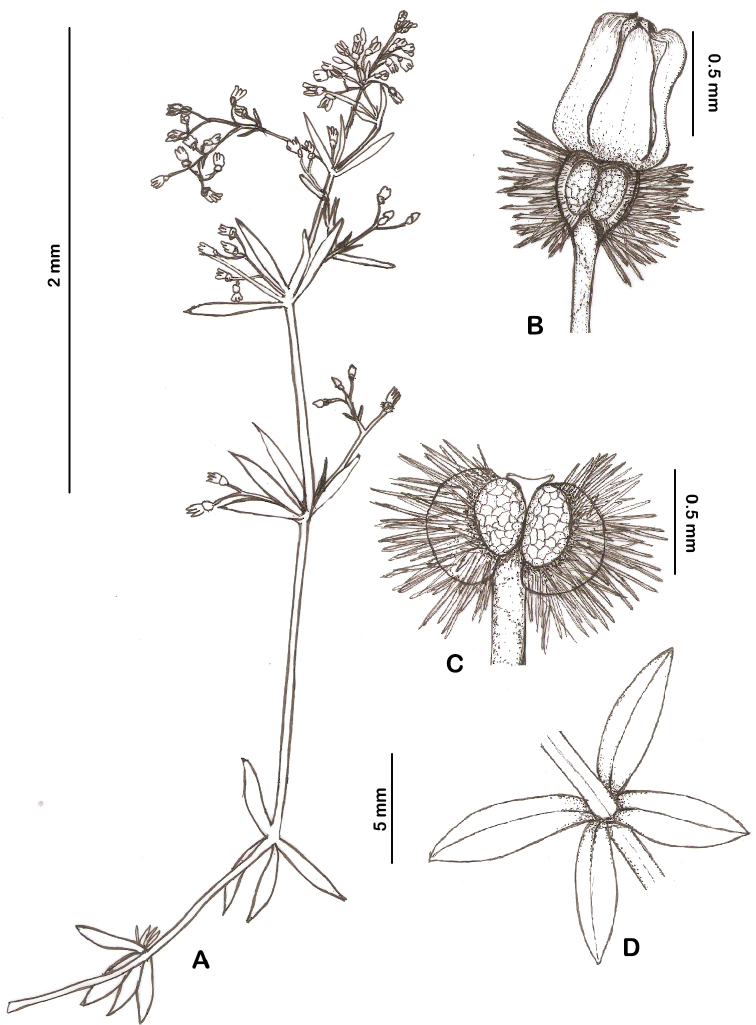
*Galium shinasii*: **A** habit **B** flower **C** fruit **D** leaves.

**Figure 2.**
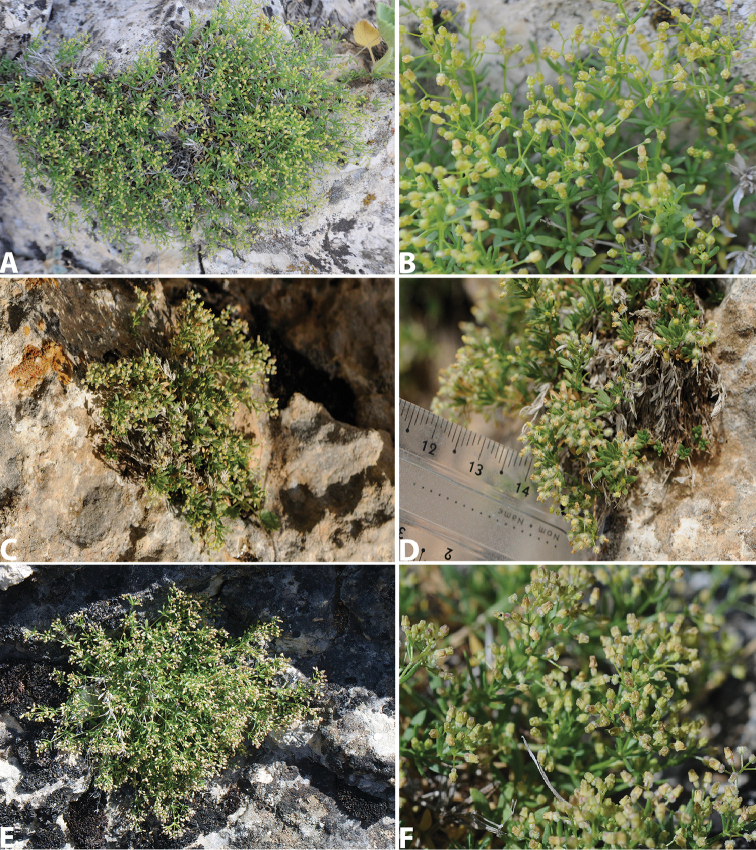
*Galium shinasii* habits: **A–B** from type locality, Levent Canyon, Malatya (H.Yıldırım 3358) **C–D** from Refahiye, Erzincan (H.Yıldırım 3694) **E–F** from Doğanşehir, Malatya (H.Yıldırım 3033).

**Figure 3.**
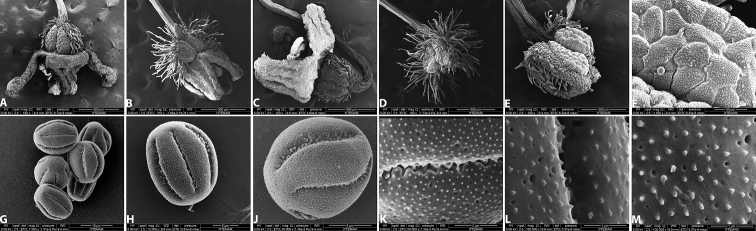
*Galium shinasii*: **A–C** flowers **D–E** fruits **F** fruit tubercles **G–J** pollen grains **K–M** detail of pollen garin surface.

#### Etymology.

This species is named in honour of retired Prof. Dr. Şinasi Yıldırımlı (Biology Dep. Hacettepe University, Turkey), who is an expert in Plant systematics and taxonomy. He described more than 100 new plant species for science in Turkey. The Turkish name of this species is given as “Levent İplikçiği”, according to the guidelines of Menemen et al. (2013).

#### Additional specimens examined.

**Figure 4.**
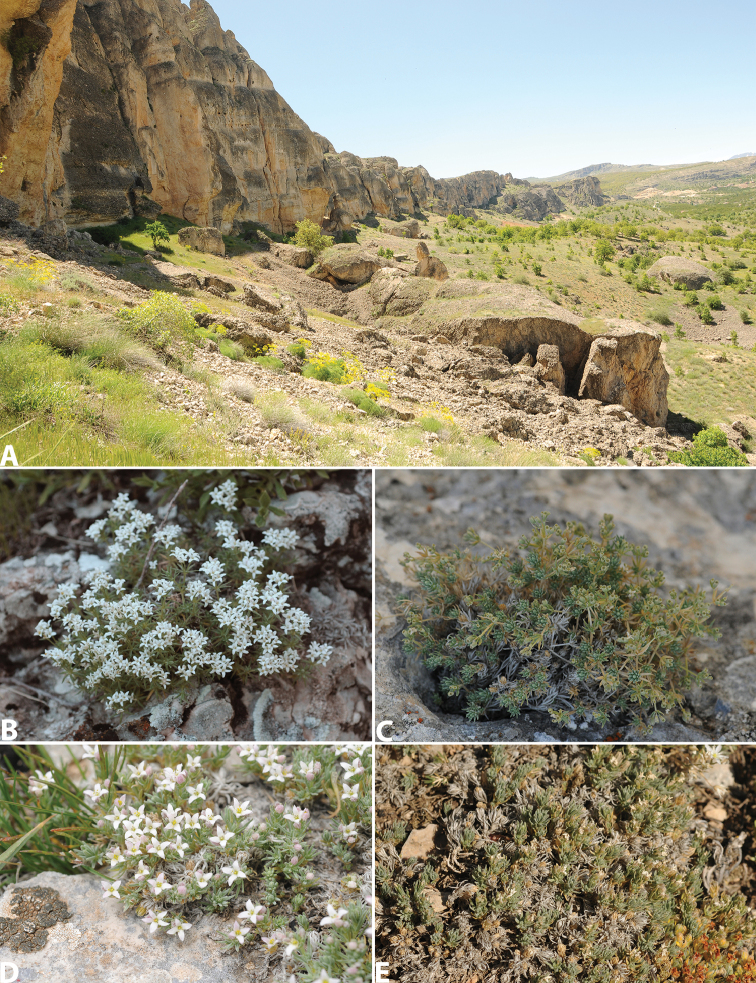
**A** type locality of *Galium shinasii*, Marlstone rocky cliffs in Levent Canyon **B–C** Galium incanum subsp. pseudocornigerum **D–F** *Galium cornigerum*

-*Galium cornigerum*: –Turkey: Malatya: Levent Kanyonu inişi, kalker kayalıklar, 30.05.2012, H.Yıldırım 2386 (EGE!); Darende, Engüzek yaylası, Akbabaçalı Dağı zirve, 2100 m, 30.05.2012. H.Yıldırım 2396 (EGE!) (Figure 4D, E).

-Galium incanum subsp. pseudocornigerum: –Turkey: Sivas: Gürün-Pınarbaşı arası, Pınarbaşı’na 68 km kala, yol kenarı kayalık alan, 1662 m, 29.06.2016, H.Yıldırım 3948 (EGE!) (Figure 4B, C).

-*Galium lasiocarpum*: –Turkey: Elazığ: in Cappadocia, Aucher 694 (holotype G!).

*Galium sorgerae*: –Turkey: Isparta: Dedegöl Da., 2200 m, 1 vii 1965, Sorger 65-42- 70 (isotype WU!).

#### Distribution and ecology.

**Figure 5.**
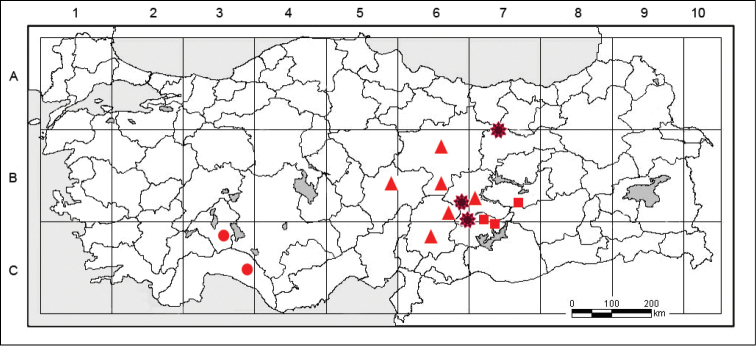
Known distribution of: *Galium shinasii* (✹), *Galium cornigerum* (▲), *Galium sorgerae* (●), *Galium lasiocarpum* (■).

*Galium shinasii* is a endemic for Eastern Anatolia. It’s known that is from Levent Canyon (Figure 5) in Akçadağ district, and Eskiköy in Doğanşehir district in Malatya province and near Refahiye district in Erzincan (Figure 5). It is an element belonging to the Irano-Turanian floristic region and colonizes only marlstone-calcareous cliffs, usually those with an eastern and south-eastern orientation, at an elevation of 1200–1800 m. It is an obligate chasmophyte.

#### Suggested conservational status.

The new species found in 3 populations. Two populations have been discovered in Malatya province, the other has been discovered in Erzincan province. Although the distribution area of *Galium shinasii* seems relatively wide, the populations of the area are very restricted. The total population area of *Galium shinasii* was calculated as 0.2 km^2^ and approximately 500 individuals were observed in total. Probably it has still several undiscovered populations. No anthropogenic or grazing effects were observed on the population. According to the present data, following the criteria laid out by the IUCN (2012), the plant is categorized as ‘Vulnerable’ (VU) D1 + 2, on account of its restricted distribution.

## Discussion and conclusions

*Galium shinasii* is a member of *Galium* Sect. *Orientigalium* Ehrend. It is characterized by chasmophyte, dwarf caespitose habit, very fragile 2–6 cm long stems, mostly 4 rarely 6 leaves in a whorl; 1.5–6.5 cm long leaves; flowers diam 1.2–1.8 mm; corolla yellowish-green to reddish-green; usually corolla lobes formed in a conical or campanulate corolla shape, very rarely lobes wholly opens and formed a infundibular corolla shape; fruit dorsal and ventral surface with densely transparent tubercles, lateral surface 0.2–0.4 mm spreading to patent hairy.

Although *Galium shinasii* shows some morphological similarities with *Galium lasiocarpum* Boiss., *Galium sorgerae* Ehrend. and Schönb., *Galium cornigerum* Boiss. and Hausk. in sect. *Orientigalium*, it is easily distinguished from these by relatively smaller flowers; yellowish-green to reddish-green and very reduced tepals; fruit surface is not only hairy on lateral surface, and also dorsal and ventral surface with densely transparent tubercles. Also it shows slight morphological similarities to *Galium incanum* Sm. subsp. *pseudocornigerum* Ehrend. with dwarf caespitose habit, smaller leaves and in having fruits lacking a calyx but it is easily distinguished from Galium incanum subsp. pseudocornigerum by its especially more reduced and different coloured flowers, a greater numbers flower number per stem; smaller, depressed subglobose and long-hairy fruits.

The detailed of the morphological differences between *Galium shinasii* and related *Galium* species are summarized in the Table 1.

**Table 1.** Main differantial characters among *Galium shinasii* and close related species *Galium sorgerae*, *Galium cornigerum* and *Galium lasiocarpum*.

Species Characters	*Galium shinasii*	*Galium sorgerae*	*Galium cornigerum*	*Galium lasiocarpum*
**Stem**	1.5–6 cm, prostrate-ascending to erect, glabrous to slightly puberulent; sometimes slightly winged on nerves,	3–4 cm, prostrate-ascending, densely hirsute	to 5 cm, prostrate-ascending, with very short, subvelutinous hairy	to 5 cm, erect to ascending, covered with straight spreading hair
**Leaves**	2–8 × 0.6–1.3 mm, linear-lanceolate to narrow elliptic; in whorls mostly 4, rarely 6 leafed	4–6 × 0.7–1 mm, linear-oblanceolate or narrowly elliptic; in whorls 6 leafed;	5–9 × 0.4–0.8 mm, linear, linear elliptic to lanceolate; in whorls 6 leafed	6–10 × 1–1.5 mm, linear-elliptic; in whorls 6 leafed
**Inflorescence**	very reduced, dicashium, mostly terminal and also axillar, 8 to 75 flowered per stem, never hidden by uppermost leaves	very reduced, few-flowered	mainly terminal reduced, corymbiform cymes, few-flowered, often ± hidden by uppermost leaves	very reduced, subumbellate-capitate, 3–8 flowered, hidden by uppermost leaves
**Pedicel**	1.5–2.5 mm, glabrous, 2–5 mm in fruiting time	2–3 mm, hirsute	0–4 mm, subvelutinous hairy	1–3 mm, hairy
**Calyx**	absent	–	2–4, subulate, persistent in fruit.	0–2
**Corolla**	yellowish-green to reddish-green; 1.2–1.8 mm diam;	whitish when dry, infundibular, 2–2.5 mm diam	white, 4–5 mm diam	white or pink, 3.5–4 mm diam
**Fruit**	dorsal and ventral surface with densely transparent tubercles, lateral surface 0.2–0.4 mm spreading to patent hairy.	hirsute	subtomentose	villous

After adding this new species in science literature, the total number of *Galium* taxa were raised to 121 (105 species) in Turkey and 61 taxa are endemic for Turkey.

## Supplementary Material

XML Treatment for Galium shinasii
